# Correction: Going with the Flow: Detection of Drift in Response to Hypo-Saline Stress by the Estuarine Benthic Diatom ***Cylindrotheca closterium***

**DOI:** 10.1371/journal.pone.0094719

**Published:** 2014-04-02

**Authors:** The PLOS ONE Staff

## Notice of Republication

This article was republished on March 17, 2014, because of errors that prevented Figure 2 from opening properly. The publisher apologizes for these errors. The originally published, uncorrected article and the republished, corrected article are provided here for reference.

## Supporting Information

File S1 Originally published, uncorrected article.

File S2 Republished corrected article.
