# Supplementary material for: Correction: Going with the Flow: Detection of Drift in Response to Hypo-Saline Stress by the Estuarine Benthic Diatom Cylindrotheca closterium
Source: PLoS One. 2014 Apr 2;9(4):e94719. doi: 10.1371/journal.pone.0094719 (PMC3973582; doi:10.1371/journal.pone.0094719)
Supplement: File S2 — Republished corrected article. [file pone.0094719.s002.pdf]

Going with the Flow: Detection of Drift in Response to Hypo-Saline Stress by the Estuarine Benthic Diatom *Cylindrotheca closterium*

Cristiano V. M. Araújo[1*], Sonia Romero-Romero[2], Lucio F. Lourençato[1], Ignacio Moreno-Garrido[2], Julián Blasco[2], Michael R. Gretz[3], Matilde Moreira-Santos[1], Rui Ribeiro[1]

1 Department of Life Sciences, University of Coimbra, IMAR-Instituto do Mar, Coimbra, Portugal, **2** Instituto de Ciencias Marinas de Andalucía (CSIC), Puerto Real, Cádiz, Spain, **3** Department of Biological Sciences, Michigan Technological University, Houghton, Michigan, United States of America

Abstract

Avoidance response is a well-known mechanism for escaping environmental stress. For organisms with reduced active movement, such as benthic microalgae, drifting could be a specifically selected mean of avoiding less favorable environments. To test this hypothesis, a system was developed to assess if hypo-saline stress triggers drift in the estuarine benthic diatom *Cylindrotheca closterium*. Concurrently, the effects of salinity on growth inhibition were also investigated in order to compare the sensitivity of this endpoint with the drift response, and to estimate the immediate population decline caused by both drift and population growth responses. It was verified that the salinity value that inhibited the algal population growth by 50% (IGS_{50}) was 19, while the salinity value that triggered the drift response by 50% of the population (TDS_{50}) was 15. These results indicate that drift is an identifiable response triggered to escape stressful environments. The combination of the two responses (population growth and drift) showed that population decline based exclusively on the inhibition of population growth may result in an underestimation of the risk, compared with the decline when drifting to avoid stress is also taken into account.

Citation: Araújo CVM, Romero-Romero S, Lourençato LF, Moreno-Garrido I, Blasco J, et al. (2013) Going with the Flow: Detection of Drift in Response to Hypo-Saline Stress by the Estuarine Benthic Diatom *Cylindrotheca closterium*. PLoS ONE 8(11): e81073. doi:10.1371/journal.pone.0081073

Editor: Jonathan H. Badger, J. Craig Venter Institute, United States of America

Received July 9, 2013; **Accepted** October 8, 2013; **Published** November 19, 2013

Copyright: © 2013 Araújo et al. This is an open-access article distributed under the terms of the Creative Commons Attribution License, which permits unrestricted use, distribution, and reproduction in any medium, provided the original author and source are credited.

Funding: This study was partially funded by the "Fundação para a Ciência e a Tecnologia" (FCT, Portugal) through a postdoctoral fellowship (reference SFRH/BPD/74044/2010) to C.V.M. Araújo, through the SALTFREE project (contract PTDC/AAC-CLI/111706/2009) and through "Ciência 2007 - Human Potential Operational Program" (POPH) and "Quadro de Referência Estratégico Nacional" (QREN) through the European Social Fund (ESF) and the Spanish Ministry of Education and Science (MEC) funds. The funders had no role in study design, data collection and analysis, decision to publish, or preparation of the manuscript.

Competing interests: The authors have declared that no competing interests exist.

* E-mail: cristiano.araujo@icman.csic.es

Introduction

A disruption in communities of microalgae can generate serious problems for the system productivity, imbalance in food webs and, in consequence, impairment of ecosystem functioning given the role played by these organisms as primary producers. Planktonic microalgae have been employed in environmental risk assessment [1] and recommended as test organisms in international guidelines [2-4] because of their ecological relevance, feasibility for cultivation and sensitivity to different contaminants [1]. More recently, following the same trend, the interest in using benthic diatoms has increased appreciably [5], and estuarine species have attracted particular interest [6-11]. The estuarine microphytobenthos community is mainly composed of sediment-inhabiting benthic diatoms, which are fundamental for estuarine systems, as they are, to a large extent, responsible for the input of energy into the system, stabilization of the sediment through the production of polysaccharides, and constitute a major food source for many species, mainly deposit-feeders [12,13]. Due to their important role in estuarine systems, the use of microphytobenthos in environmental risk assessment has been strongly encouraged [14]. However, in practice, the use of benthic diatoms in toxicity testing has been limited to estimations of population growth (e.g. see references in Araújo et al. [14]). Being motile organisms, diatoms exhibit measurable behavioral responses associated with their displacement [15]; these responses may be of interest for ecotoxicological testing but have been neglected in this context.

The intriguing, sophisticated and complex horizontal displacement mechanism of benthic diatoms has been explored over many years [16]. Besides this typical displacement, benthic diatoms are also able to perform vertical displacement in the sediment in response to environmental

gradients of light, nutrients, salinity, and temperature [17-19]. Although, in general, studies about diatom displacement have not focused specifically on habitat disruption scenarios, the displacement velocity of the benthic diatom *Craticula cuspidata* has been shown to be a good indicator of the toxicity of freshwater sediment elutriates [17]. Therefore, it is possible to hypothesize that motility reactions could occur as an active response to avoid contamination. Recently, avoidance by swimming to escape less favorable contaminated environments has received special attention, justified by the ecological relevance of the results with different invertebrate and vertebrate species [20-23]. Such a response may be very significant for the immediate decline of a population, as it can be observed in the short-term at sub-lethal contamination levels, with consequences ecologically similar to the death of the population [23,24]. For microalgae, the most common approach to assess contaminant effects on their displacement ability does not take into account a potential avoidance response, but rather the use of parameters related to their innate motility (e.g. percentage of swimming cells, velocity and distance), such as the Erlanger flagellate test with the freshwater green alga *Euglena gracilis* [25]. The displacement of microalgae by swimming was recently documented in the planktonic marine golden brown algae *Heterosigma akashiwo*, but in this case to escape predation [26]. Nevertheless, given that, in the case of benthic diatoms, active displacement is not expected to enable cells to move long distances; self-propelled (active) avoidance of contamination would not be an effective response to the detected contamination. Therefore, drift, characterized by the displacement of the organisms by means of the water flow [27], may be anticipated as an alternative mechanism for microalgae to move sufficiently long distances.

Drift has been widely recorded in freshwater macroinvertebrates in response to predation, population density, life-cycle stage, food availability and quality, water flow/discharge, photoperiod, and water chemistry [27,28]. Additionally, this type of displacement has also been verified for organisms exposed to contaminants, especially agrochemicals [29-31]. To date, drift has not been recorded for benthic diatoms, but changes from horizontal to vertical movement patterns were verified in the benthic diatom *Cylindrotheca closterium* when exposed to extreme salinity and nutrient conditions [15]. These authors observed that, under optimal conditions, the typical movement presented by that species was gliding (their innate horizontal pattern); however, under dramatic hypo-saline conditions, changes from gliding to pivot and pirouette movements (vertical patterns) were recorded. This movement by cells (interpreted as an attempt to detach themselves from sediment) could therefore be an indication of their potential for transportation. Given the low active mobility of *C. closterium* (velocity up to 840 µm min^{-1} [32]), its ability to change the movement pattern from horizontal to vertical under stressful conditions (e.g. salinity) [15], as well as the evidence that the spatial distribution of estuarine benthic diatoms can be strongly salinity-driven [33], we hypothesized that *C. closterium* may use drift as an avoidance response to escape less favorable environments.

The main goal of the present study, therefore, was to verify the drift response of *C. closterium* to avoid potentially stressful (decreased) salinity conditions. Classical population growth inhibition assays were carried out together with drift assays to verify how *C. closterium* population growth is inhibited by decreases in salinity; and, on this basis, both growth and drift responses have been combined to estimate a toxicity parameter that is more relevant in ecological terms – the immediate population decline. Salinity was used as stress factor as important changes in salinity values in estuaries due to, for instance, extreme weather events (intense drought or rainfall) can cause disruption in the habitats and biological communities [34].

Materials and Methods

Ethics statement: for water sampling in Río San Pedro, specific permission is not required because this is a public beach zone; the field studies did not involve endangered or protected species.

Test organism

A strain of *C. closterium* (Ehrenberg) Lewin and Reimann cultured for more than ten years in the Marine Sciences Institute of Andalusia (ICMAN-CSIC, Spain) and included in the Culture Collection of Marine Microalgae of the ICMAN (CCMM-ICMAN, BIOCISE) was used as test organism. Seawater sampled in the Río San Pedro (SW, Spain) was filtered (GF/C Whatman filter), autoclaved, and later enriched with Guillard's f/2 medium [35] and 50 mg L^{-1} of SiO^2, to be used as culture medium. Cultures were maintained at 20 ± 1 °C under continuous white light (35.2 ± 1.1 µmol quantum m^{-2} s^{-1}) in a controlled culture chamber (Ibercex). Cultures in exponential growth (4 to 7 days old) were used in the experiments.

Salinity treatments

Salinity treatments were prepared from the culture medium by diluting it with Milli-Q water (pH of 6.15) enriched with nutrients in the same quantities as the culture medium. In all experiments the salinity (HANNA Instruments, Seawater Refractometer HI 96822) value of the control (non-diluted culture medium) was between 37 and 35 (pH around 8.2), whereas in the different salinity treatments the value varied from 30 to 0 (pH ranged from 8.01 to 7.78 at salinities 30 to 5).

Test system for drift assays

The test system used in the drift assays was formed by three interconnected compartments (transparent polypropylene plastic flasks; 35 mL each) and a recirculation reservoir (1 L capacity, but filled with 500 mL), forming a closed flow system (Figure 1). The first and second compartments (respectively at the closed end and in the middle) were both designed to reduce water turbulence and prevent chaotic drift; the first was used as water entry chamber and the second to smooth the flow and make it more laminar. The third compartment (at the open end to allow the water to flow out) was designed as the exposure chamber, i.e., the compartment containing the microalgae cells. The water flow was maintained at 440 ± 13

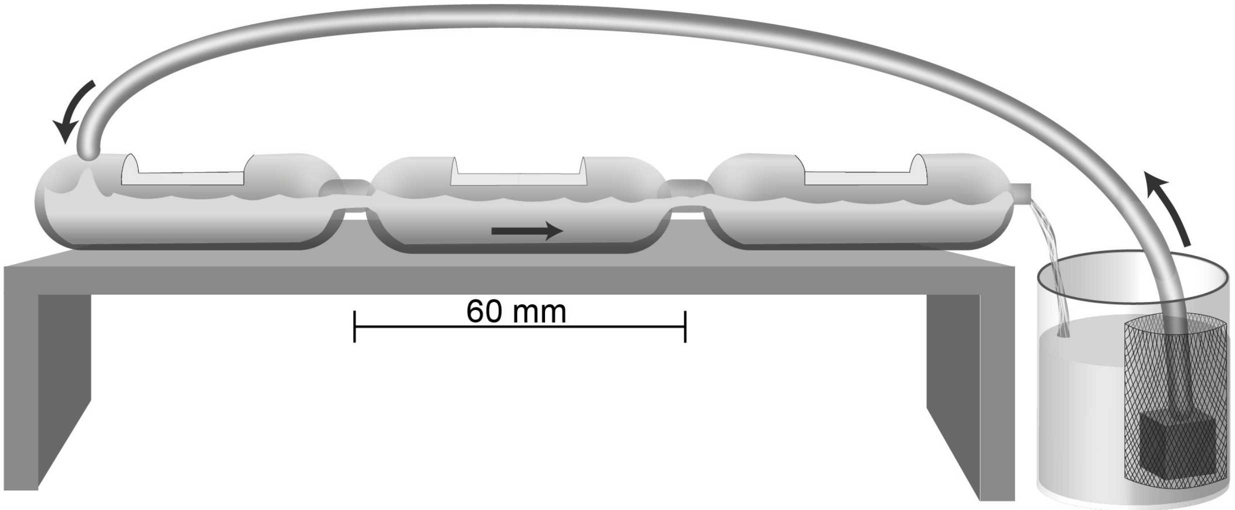

Figure 1. Scheme of the three-compartment assay chamber to evaluate drift by the benthic estuarine microalgae *Cylindroteca closterium.*
doi: 10.1371/journal.pone.0081073.g001

mL min^{-1} by a pump placed in the recirculation reservoir. The pump was wrapped in a mesh of 5 µm to block the reintroduction of the drifted microalgae into the system.

Growth assay

A 72-h growth assay was performed following Moreno-Garrido et al. [7]; a shorter time is not able to discriminate differences in the growth population. Six salinity treatments (30, 24, 19, 12, 5, and 0) plus a control (culture medium with no change in salinity) were prepared as described above and assayed in triplicate in 125-mL Erlenmeyer flasks. Each flask was filled with 50 mL solution and stoppered with synthetic cotton (Perlon). The initial cell concentration was $1x10^4$ cells mL^{-1} (a volume of less than 50 µL of cell suspension was used as inoculum to prevent changes in salinity). All treatment cultures were incubated under controlled light and temperature conditions similar to those of the stock cultures. After the 72-h exposure, cell numbers were counted under the microscope using a Neubauer chamber, to estimate the assay endpoint as growth inhibition percentages relative to the control treatment (taken as the maximum growth) [9].

Drift assay

For the 12-h drift assay, six salinity treatments (9,12,15,19,24,30) plus a control treatment, quadruplicated in time, were assayed. The minimum salinity level chosen was 9 because represented the salinity at which growth was inhibited by 95% (IGS95); at lower salinities the displacement ability of the cells could be damaged [15] and cells starting to die (inhibition growth higher than 100%). The remaining salinity levels corresponded to the IGS10, IGS25, IGS50, IGS75 and IGS90. For each treatment, a volume of 500 mL was placed in the water reservoir with its respective salinity. A concentrated cell suspension of *C. closterium* was introduced into the third compartment, at a concentration of $1x10^4$ cells mL^{-1} relative to the total volume of water (500 mL; a volume of ca. 500 µL of cell suspension was used as inoculum to prevent dilution of the system salinity). Cells were kept in the compartment in darkness for 6 h to settle and to allow adhesion. After this period, the pump was started and maintained for the 12-h exposure period. Assays were performed at the same temperature as the growth assay and in darkness to prevent cell multiplication. At the end of each assay, the pump was stopped and the compartment containing the microalgae was isolated using plasticine plugs wrapped in parafilm. The microalgal suspension in that compartment was homogenized and 12 sub-samples (300 µL) were taken to determine cell numbers, i.e., to count the cells that had not drifted, indirectly measured (microplate reader, Tecan Infinite F200) by chlorophyll fluorescence (a reliable indicator of cell density: $r=0.968$; $p<0.0001$; $n=13$).

To fully evaluate the drift response of *C. closterium* with the proposed system design, i.e., to be sure that all cells not found at the end of the assay were drifters, two preliminary experiments were set up to investigate for possible confounding factors. The first experiment intended to verify the possible occurrence of cell growth or mortality during the 12-h exposure period in darkness. It was conducted in tubes with 5 mL volume using a cell density of $10x10^4$ cells mL^{-1} exposed to the same salinity treatments in order to detect cell growth or death after 12 h exposure. A higher cell density was used so that cells could be counted using a Neubauer chamber and a cell density $1x10^4$ cells mL^{-1} is the threshold for a precise count. Given that no differences in cell density were observed among the eight treatments at the end of the exposure period, it was concluded that neither growth nor mortality occurred at the tested salinities during the 12 h exposure in darkness (one-way

Table 1. Mean cell density (x10^4 cells mL^{-1}) and standard deviation (SD; n = 3) at the start and end (12 h) of the preliminary experiment for verifying the occurrence of cell growth or mortality at the different salinities during 12-h exposures in darkness.

Salinity		Cell density	SD
Start	37	10	2.5
End	37	11.7	0.7
	30	11.7	0.8
	24	12.3	1.9
	19	12.5	1.2
	15	12.8	0.4
	12	12.2	0.8
	9	10.3	0.5

doi: 10.1371/journal.pone.0081073.t001

analysis of variance [ANOVA]: $F_{7,16}$ = 0.6918; p>0.05) (Table 1).

The second experiment intended to verify the possible occurrence of chaotic drift due to the initial water turbulence associated with the restarting of the pump after the 6 h period for the settling and adhesion of cells. An experiment using only control medium was set up following exactly the same procedures described above for the drift assay. However, after the 6-h settling and adhesion period, the pump was re-started for only 30 s, since after this period the system stabilizes. The drift observed during this 30-s period was considered chaotic drift caused by the initial system turbulence. Cell counting was performed under the microscope using a Neubauer chamber. Given that only a mean (± standard deviation [SD)]) percentage of 3% (± 5; n = 7) of the cells initially introduced into the system drifted chaotically, the initial turbulence of the flow in the system was considered negligible.

Data analysis

The number of cells that drifted was calculated by the difference between the number of cells initially introduced into the third compartment and the number of cells remaining in the same compartment after the 12-h exposure. Statistical differences in growth and drift responses between the different salinities were verified by one-way ANOVA followed by the Dunnett´s multiple comparison test. The IGS_{50} (inhibition growth salinity - salinity value at which a 50% inhibition of cell growth was observed) and TDS_{50} (total drift salinity - salinity value that 50% of the cells drifted) and corresponding 95% confidence intervals (CI) were calculated using PriProbit 1.63 software [36]. The immediate population decline caused by the different salinities was estimated by summing the percentage of drifter cells and the percentage of growth inhibition of the remaining cells (non-drifters). Considering drift in the control as a natural drift rate, the salinity value that causes a population decline of 50% was calculated using model 2 of the PriProbit 1.63 software. Salinity values used to calculate the population decline were those used in the drift assay; therefore, when a salinity level was not assayed in the growth inhibition assay its effect was estimated using the PriProbit 1.63 software (i.e., salinity of 15 = IGS_{75} and salinity of 9 = IGS_{95}).

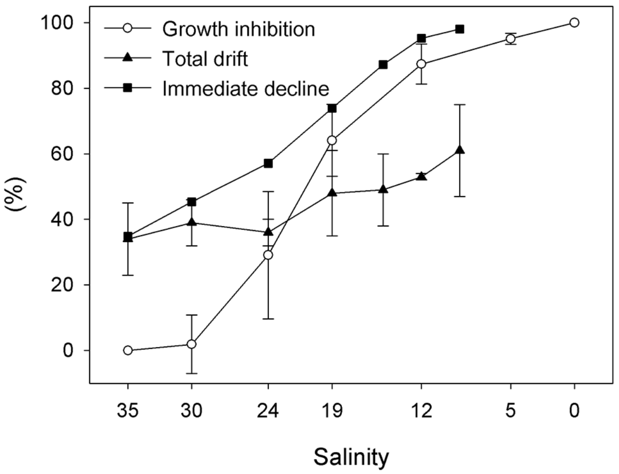

Figure 2. Mean percentage of growth inhibition (n = 3), total drift (n = 4) and immediate population decline of the benthic microalgae *Cylindrotheca closterium* exposed to different salinities. Data of immediate decline represent the sum of 12-h drift percentages and 72-h growth inhibition percentages of the remaining cells.
Error bars indicate + 1 standard deviation. Statistical differences in responses relative to the control are indicated by * (p<0.05) and ** (p<0.01) according to the Dunnett's multiple comparison test.

doi: 10.1371/journal.pone.0081073.g002

Results

Growth assay

Significant differences in growth inhibition were found among salinity values (one-way ANOVA: $F_{6,14}$ = 61.993, p < 0.0001), but a significant inhibition in growth relative to the control was found only from salinities 24 to 0 (Dunnett´s test: p < 0.05). Whereas in the control treatment the cell density increased 8.6 times during the assay, cell growth was non-existent at salinities as low as 12, with final cell densities equal to the initial. At the two lowest salinity values (5 and 0) the final cell number was lower than the initial cell number. Taking the cell density in the control treatment as maximum growth (100%), the growth inhibition percentage for different salinities tested ranged between 2.2 and 113% (Figure 2). For salinities equal to or lower than 19 the growth inhibition was higher than 50%. The 72-h IGS_{50} salinity value was 19 (95% CI: ±4).

Drift assay

Total drift occurred at all salinities, including the control treatment, and ranged from around 30 to 60%, increasing with decreasing salinity (Figure 2). Drift in control was around 30% and it was very similar at salinity 30 and 24. At salinities equal to or lower than 19, drift was close to or higher than 50%, and reached more than 60% at salinity 9. Although significant

differences in drift were found among the various salinities (one-way ANOVA: $F_{6,20} = 3.702$, $p = 0.0123$), only at the lowest salinity value was drift significantly higher than in the control (Dunnett´s test: $p < 0.01$). The salinity value at which 50% of cells drifted (TDS_{50}) was 15 (95% CI: 12–18).

From the results of the 72-h growth inhibition and 12- h drift assays, the immediate population decline was estimated for all salinity levels used in drift assays (Figure 2). The salinity value at which there was a population decline of 50% due to growth inhibition and drift, regarding the control, was 24 (95% CI: 23–28).

Discussion

The ability of the estuarine benthic diatom *C. closterium* to employ drifting to avoid stressful conditions caused by a decrease in salinity has been studied. Although effects on population growth have previously been the most frequently-used endpoint in toxicity testing, drift may also have important effects on the population dynamics of benthic diatoms, as a mechanism for avoiding a less favorable area and, consequently, colonizing other areas or, afterwards, the same area upon habitat recovery. In addition, both responses acting simultaneously can accelerate effects on the population decline, although drift has more immediate consequences. At salinity 24, for instance, the *C. closterium* population had its growth inhibited by 33% (IGS_{33}), while at this salinity the estimated drift was around 36%, slightly higher than control. Therefore, a decrease measured in population growth should be estimated taking into account ca. 64% of the non-drifter population. On the other hand, with a reduction in salinity to 19, growth was inhibited by 60% and drift was around 50%. This most extreme stress scenario highlights the significant effect that drift can have on the maintenance of the population of benthic diatoms, and the potential effects of drift on local population decline. The drift results obtained in the present study were to some extent expected, since diatoms are organisms very capable of responding to environmental physicochemical alterations, and they can use complex mechanisms to execute their response [37]. Among their behavioral changes, movement-related parameters have been widely studied [17-19,38,39].

In particular, movement responses to stressful environmental factors can be the result of an adaptation that allows cells to inhabit highly variable environments [17]. Sauer et al. [19] showed that vertical migratory movements of diatom assemblages were influenced by light and salinity. Also, light- and grain size-dependent vertical motility was observed in *C. closterium* [40]. Vertical migration behavior was also demonstrated in function of the nutrient levels in subsurface sediments [18]. McLachlan et al. [39] did not find a phototactic response in *C. closterium*, whereas that response was observed in the estuarine benthic microalgae *Navicula perminuta*. Temperature has also been shown to be a factor able to change cell speed in horizontal movements of diatoms, which was reduced at 40 °C [41]. Particularly in estuarine systems, sediment-inhabiting diatoms are propelled to move constantly due to the dynamics of these systems and, in this context, salinity therefore has a strong influence on the displacement of benthic diatoms, as a factor controlling migration [19,42,43].

According to Apoya-Horton et al. [15], movement modalities, referred to as pirouettes and pivots, exhibited by *C. closterium* when exposed to suboptimal conditions may be mechanisms adopted by individual cells to detach themselves from sediment and thus avoid longer exposure to those conditions. Those movements could indicate that cells are demonstrating a propensity to perform drifting. Although this interpretation cannot be considered certain, the ability of *C. closterium* to drift (rather than to remain attached to a grain of sediment) under stressful conditions is obvious.

There is additional value in studying the way drift is used as measurable avoidance response by benthic diatoms, since such an approach does not focus exclusively on variation in the type or velocity of the cell's activity/movement as its response to a stressful environment. This response represents displacement activity that is both reliable and very relevant for studying the effects of environmental disruption on the spatial distribution of benthic diatom populations. In an ecotoxicological context, data about the impairment of microalgae motility is scarce [44]. The latter authors showed that the motility of the marine microalgae *Isochrysis galbana* and *Tetraselmis chui* is a rapid and sensitive endpoint for assessing metals toxicity. Motility in the benthic diatom *Craticula cuspidata* was also reduced when exposed to potentially toxic elutriates [17]. Measurement of the motility and orientation of the flagellate green microalgae *Euglena gracilis* was shown to be effective for assessing exposure to toxic compounds [25]. Measuring the distance travelled by free-swimming microalgae may be a more accurate indicator of active avoidance; and recently it was shown that the microalga *H. Akashiwo* presented active avoidance behavior when exposed to predators [26]. Although vertical movement response typically studied in benthic diatoms can contribute to a better understanding of temporal and spatial patterns of diatom distribution [41], in the scenario of a large spatial contamination gradient, this type of response may not achieve effective avoidance; therefore the horizontal displacement achieved by means of drift could be an alternative response by which benthic diatoms move away from contamination by sufficiently long distances.

It can be difficult to discriminate separately the effects of salinity from those of other environmental disruptors (e.g. contaminants). Our results indicate that *C. closterium* cells can grow at salinity values lower than 35-37, i.e., at values close to 30, at which growth was optimal. However at salinities of 24 and less, growth will be inhibited, and simultaneously, local populations can be further reduced as a result of drift and not necessarily due to direct toxic effects. Although being a largely neglected perspective in ecotoxicity studies, contaminants can act as habitat disruptors, affecting population dynamics, community structure and ecosystem functioning with no effect whatsoever at the individual level of biological organization [45,46]. Thus, although determining the main factors that give rise to drift by microphytobenthos is an important finding, it is equally relevant to understand and quantify the effects that this

displacement mechanism can generate on natural population and the consequences for ecosystem structure and functioning.

Conclusions

Drift response by the estuarine benthic diatom microalgae *C. closterium* was detected in response to hypo-saline stress. Although the inhibition of population growth as a standard response was as sensitive as drift, the combination of both responses for estimating the real salinity effect on the immediate population decline showed that the drift response of *C. closterium* should not be neglected under such stressful conditions.

Acknowledgements

Assistance with the graphics of Figure 1 was provided by Nadia Romero.

Author Contributions

Conceived and designed the experiments: CA SRR LFL IMG JB MRG MMS RR. Performed the experiments: CA SRR LFL. Analyzed the data: CA SRR LFL IMG JB MMS RR. Contributed reagents/materials/analysis tools: IMG JB MRG MMS RR. Wrote the manuscript: CA SRR LFL IMG JB MRG MMS RR.

References

1. Torres MA, Barros MP, Campos SCG, Pinto E, Rajamani S et al. (2008) Biochemical biomarkers in algae and marine pollution: A review. Ecotoxicol Environ Saf 71: 1-15. doi:10.1016/j.ecoenv.2008.05.009. PubMed: 18599121.
2. ISO 8692 (1989) Water quality; —— fresh water algal growth inhibition test with *Scenedesmus subspicatus* and *Selenastrum capricornutum*. Geneva, Switzerland: International Organization for Standardization.
3. OECD, Organization for Economic Cooperation and Development (1998) OECD guideline for testing of chemicals: algae growth inhibition test. Paris: OECD Publications.
4. USEPA (2002) Short-term methods for estimating the chronic toxicity of effluents and receiving waters to freshwater organisms. U.S. Environmental Protection Agency, EPA-821-R-02-013.
5. Larras F, Bouchez A, Rimet R, Montuelle B (2012) Using bioassays and species sensitivity distributions to assess herbicide toxicity towards benthic diatoms. PLOS ONE 7: e44458. doi:10.1371/journal.pone.0044458. PubMed: 22952981.
6. Stauber JL, Florence TM (1985) The influence of iron on copper toxicity to the marine diatom, *Nitzschia closterium* (Ehrenberg) W. Smith. Aquat Toxicol 6: 297-305. doi:10.1016/0166-445X(85)90025-6.
7. Moreno-Garrido I, Hampel M, Lubián LM, Blasco J (2003) Sediment toxicity tests using benthic marine microalgae *Cylindrotheca closterium* (Ehremberg) Lewin and Reimann (Bacillariophyceae). Ecotoxicol Environ Saf 54: 290-295. doi:10.1016/S0147-6513(02)00077-5. PubMed: 12651184.
8. Moreno-Garrido I, Lubián LM, Jiménez B, Soares AMVM, Blasco J (2007) Estuarine sediment toxicity tests on diatoms: sensitivity comparison for three species. Estuar Coastal Shelf Sci 71: 278-286. doi:10.1016/j.ecss.2006.08.003.
9. Araújo CVM, Diz FR, Laiz I, Lubián LM, Blasco J et al. (2009) Sediment integrative assessment of the Bay of Cádiz (Spain): an ecotoxicological and chemical approach. Environ Int 35: 831-841. doi:10.1016/j.envint.2009.02.003. PubMed: 19318227.
10. Araújo CVM, Tornero V, Lubián LM, Blasco J, van Bergeijk SA et al. (2010) Ring test for whole-sediment toxicity assay with a benthic marine diatom. Sci Total Environ 408: 822-828. doi:10.1016/j.scitotenv.2009.10.018. PubMed: 19906403.
11. Mauffret A, Moreno-Garrido I, Blasco J (2010) The use of marine benthic diatoms in a growth inhibition test with spiked whole-sediment. Ecotoxicol Environ Saf 73: 262-269. doi:10.1016/j.ecoenv.2009.12.001. PubMed: 20031213.
12. MacIntyre HL, Geider RJ, Miller DC (1996) Mycrophytobenthos: the ecological role of the “secret garden” of unvegetated, shallow-water marine habitats. I. Distribution, abundance and primary production. Estuarines 19(2A): 186-201.
13. Miller DC, Geider RJ, MacIntyre HL (1996) Mycrophytobenthos: the ecological role of the “secret garden” of unvegetated, shallow-water marine habitats. II. Role in sediment stability and shallow-water food webs. Estuarines 19(2A): 202-212.
14. Araújo CVM, Blasco J, Moreno-Garrido I (2010) Microphytobenthos in ecotoxicology: a review of the use of marine benthic diatoms in bioassays. Environ Int 36: 637-646. doi:10.1016/j.envint.2010.04.017. PubMed: 20493528.
15. Apoya-Horton MD, Yin L, Underwood GJC, Gretz MR (2006) Movement modalities and response to environmental changes of the mudflat diatom *Cylindrotheca closterium* (Bacillariophyceae). J Phycol 42: 379-390. doi:10.1111/j.1529-8817.2006.00194.x.
16. Drum RW, Hopkins JT (1966) Diatom locomotion: an explanation. Protoplasma 62: 1-33. doi:10.1007/BF01254629.
17. Cohn SA, McGuire Jr (2000) Using diatom motility as an indicator of environmental stress: effect of toxic sediment elutriates. Diatom Res 15: 19-29. doi:10.1080/0269249X.2000.9705484.
18. Kingston MB (2002) Effect of subsurface nutrient supplies on the vertical migration of *Euglena proxima* (Euglenophyta). J Phycol 38: 872-880. doi:10.1046/j.1529-8817.2002.t01-1-01197.x.
19. Sauer J, Wenderoth K, Maier UG, Rhiel E (2002) Effects of salinity, light and time on the vertical migration of diatom assemblages. Diatom Res 17: 189-203. doi:10.1080/0269249X.2002.9705538.
20. Lopes I, Baird DJ, Ribeiro R (2004) Avoidance of copper contamination by field populations of *Daphnia longispina*. Environ Toxicol Chem 23: 1702-1708. doi:10.1897/03-231. PubMed: 15230322.
21. Moreira-Santos M, Donato C, Lopes I, Ribeiro R (2008) Avoidance tests with small fish: determination of the median avoidance concentration and of the lowest-observed-effect gradient. Environ Toxicol Chem 27: 1575-1582. PubMed: 18260687.
22. Rosa R, Moreira-Santos M, Lopes I, Picado A, Mendonça F et al. (2008) Development and sensitivity of a 12-h laboratory test with *Daphnia magna* Straus based on avoidance of pulp mill effluents. Bull Environ Conta Toxicol 81: 464-469. doi:10.1007/s00128-008-9538-y. PubMed: 18779915.
23. Rosa R, Materatski P, Moreira-Santos M, Sousa JP, Ribeiro R (2012) A scaled-up system to evaluate zooplankton spatial avoidance and population immediate decline concentration. Environ Toxicol Chem 31: 1301-1305. doi:10.1002/etc.1813. PubMed: 22467353.
24. Araújo CVM, Shinn C, Moreira-Santos M, Lopes I, Espíndola ELG, Ribeiro R (2013) Copper-driven avoidance and mortality by temperate and tropical tadpoles. Aquat Toxicol. doi:10.1016/j.aquatox.2013.10.030.
25. Häder D-P, Lebert M, Tahedl H, Richter P (1997) The Erlanger flagellate test (EFT): photosynthetic flagellates in biological dosimeters. J Photochem Photobiol B. Biology 40: 23-28. doi:10.1016/S1011-1344(97)00028-6.
26. Harvey EL, Menden-Deuer S (2012) Predator-induced fleeing behavior in phytoplankton: a new mechanism for harmful algal bloom formation? PLOS ONE 7: e46438. doi:10.1371/journal.pone.0046438. PubMed: 23029518.
27. Brittain JE, Eikeland TJ (1988) Invertebrate drift – a review. Hydrobiologia 166: 77-93. doi:10.1007/BF00017485.
28. Flecker AS (1992) Fish predation and the evolution of invertebrate drift periodicity: evidence from neotropical streams. Ecology 73: 438-448. doi:10.2307/1940751.
29. Hose GC, Lim RP, Hyne RV, Pablo F (2002) A pulse of endosulfan-contaminated sediment affects macroinvertebrate in artificial stream. Ecotoxicol Environ Saf 51: 44-52. doi:10.1006/eesa.2001.2127. PubMed: 11800549.
30. Lauridsen RB, Friberg N (2005) Stream macroinvertebrate drift response to pulsed exposure of the synthetic pyrethroid lambda-cyhalothrin. Environ Toxicol 20: 513-521. doi:10.1002/tox.20140. PubMed: 16161113.
31. Betekov MA, Liess M (2008) Potential of 11 pesticides to initiate downstream drift of stream macroinvertebrates. Arch Environ Contam Toxicol 55: 247-253. doi:10.1007/s00244-007-9104-3. PubMed: 18180861.

32. Moreno-Garrido I, Araújo CVM, Blasco J (2009) Microphytobenthos. In WT KerseySP Munger. Marine Phytoplankton. Oceanography and Ocean Engineering Series. Nova Science Publishers, Inc. New York. pp. 1-44.
33. Underwood GJC, Phillips J, Saunders K (1998) Distribution of estuarine benthic diatom species along salinity and nutrient gradients. Eur J Phycol 33: 173-183. doi:10.1080/09670269810001736673.
34. Cardoso PG, Raffaelli D, Pardal MA (2008) The impact of extreme weather events on the seagrass *Zostera noltii* and related *Hydrobia ulvae* population. Mar Pollut Bull 56: 483-492. doi:10.1016/j.marpolbul.2007.11.006. PubMed: 18164733.
35. Guillard RRL, Ryther JH (1962) Studies on marine planktonic diatoms. I. *Cyclotella nana* Hustedt and *Detonula confervaceae* (Cleve) Gran. Can J Microbiol 8: 229-239. doi:10.1139/m62-029. PubMed: 13902807.
36. Sakuma M (1998) Probit analysis of preference data. Appl Entomol Zool 33: 339-347.
37. Falciatore A, d'Alcalà MR, Croot P, Bowler C (2012) Perception of environmental signals by a marine diatom. Science 288: 2363-2366. PubMed: 10875921.
38. Cohn SA, Bahena M, Daves JT, Ragland RL, Rauschenberg CD et al. (2004) Characterisation of the diatom photophobic response to high irradiance. Diatom Res 19: 167-179. doi:10.1080/0269249X.2004.9705869.
39. McLachlan DH, Brownlee C, Taylor AR, Geider RJ, Underwood GJC (2009) Light-induced motile response of the estuarine benthic diatoms *Navicula perminuta* and *Cylindrotheca closterium* (Bacillariophyceae). J Phycol 45: 592-599. doi:10.1111/j.1529-8817.2009.00681.x.
40. Du GY, Oak JH, Li H, Chung IK (2010) Effect of light and sediment grain size on the vertical migration of benthic diatoms. Algae 25: 133-140. doi:10.4490/algae.2010.25.3.133.
41. Cohn SA, Farrell JF, Munro JD, Ragland RL, Weitzell RE et al. (2003) The effect of the temperature and mixed species composition on diatom motility and adhesion. Diatom Res 18: 225-243. doi:10.1080/0269249X.2003.9705589.
42. Clavero E, Hernández-Mariné M, Grimalt JO, Garcia-Pichel F (2000) Salinity tolerance of diatoms from thalassic hypersaline environments. J Phycol 36: 1021-1034. doi:10.1046/j.1529-8817.2000.99177.x.
43. Underwood GJC, Provot L (2000) Determining the environmental preference of four estuarine epipelic diatom taxa: growth across a range of salinity, nitrate and ammonium conditions. Eur J Phycol 35: 173-182. doi:10.1080/09670260010001735761.
44. Liu G, Chai X, Shao Y, Hu L, Xie Q et al. (2011) Toxicity of copper, lead, and cadmium on the motility of two marine microalgae *Isochrysis galbana* and *Tetraselmis chui*. J Environ Sci (China) 23: 330-335. doi:10.1016/S1001-0742(10)60410-X. PubMed: 21517009.
45. Dickman CR, Pimm SL, Cardillo M (2007) The pathology of biodiversity loss: the practice of conservation. In: DW MacdonaldK Service. Key Topics in Conservation Biology. Blackwell Publishing, Malden, MA, USA pp 1–16.
46. Ribeiro R, Lopes I (2013) Contaminant driven genetic erosion and associated hypotheses on alleles loss, reduced population growth rate and increased susceptibility to future stressors – an essay. Ecotoxicology 22: 889-899. doi:10.1007/s10646-013-1070-0. PubMed: 23604582.
